# sGC stimulation lowers elevated blood pressure in a new canine model of resistant hypertension

**DOI:** 10.1038/s41440-021-00748-5

**Published:** 2021-09-22

**Authors:** Julia Vogel, Philip Boehme, Susanne Homann, Mario Boehm, Katharina Andrea Schütt, Katharina Boden, Jakob Balitzki, Jörg Hüser, Wilfried Dinh, Hubert Truebel, Peter Sandner, Thomas Mondritzki

**Affiliations:** 1 Bayer AG, Wuppertal, Germany; 2 University of Witten/Herdecke, Witten, Germany; 3 University of Duisburg-Essen, Essen, Germany; 4 Heinrich-Heine-University, Düsseldorf, Germany; 5 Universities of Giessen and Marburg Lung Center (UGMLC), Justus-Liebig-University Giessen, German Center for Lung Research (DZL), Giessen, Germany; 6 Department of Internal Medicine I, University Hospital RWTH Aachen, Aachen, Germany; 7 Hannover Medical School, Hannover, Germany; 8 Department of Cardiology, HELIOS University Clinic Wuppertal, Wuppertal, Germany

**Keywords:** Animal model, Hypertension, sGC stimulator, BAY 41-2272

## Abstract

Therapy-resistant hypertension is a serious medical problem, causing end-organ damage, stroke, and heart failure if untreated. Since the standard of care fails in resistant hypertension patients, there is still a substantial unmet medical need for effective therapies. Active stimulation of soluble guanylyl cyclase via novel soluble guanylyl cyclase stimulators might provide an effective treatment option. To test this hypothesis, we established a new experimental dog model and investigated the effects of the soluble guanylyl cyclase-stimulator BAY 41-2272. In beagle dogs, a resistant hypertension phenotype was established by combining unilateral renal wrapping with the occlusion of the renal artery in the contralateral kidney. The most frequently used antihypertensive drugs were administered orally, either alone or in combination, and their acute effect on telemetric measured blood pressure was assessed and compared with that of BAY 41-2272. The chosen disease stimulus led to a moderate and stable increase in blood pressure. Even high doses of standard-of-care antihypertensives only slightly decreased blood pressure. In contrast, the administration of the soluble guanylyl cyclase stimulator BAY 41-2272 as standalone therapy led to a dose-dependent reduction in blood pressure (−14.1 ± 1.8 mmHg). Moreover, BAY 41-2272 could also further decrease blood pressure in addition to a triple combination of standard-of-care antihypertensives (−28.6 ± 13.2 mmHg). BAY 41-2272 was highly efficient as a standalone treatment in resistant hypertension but was also effective in addition to standard-of-care treatment. These data strongly suggest that soluble guanylyl cyclase stimulators might provide an effective pharmacologic therapy for patients with resistant hypertension.

## Introduction

Hypertension (HTN) is the most common risk factor for cardiovascular (CV) events such as stroke or heart failure [1]. It is well known that effective blood pressure (BP) lowering can prevent CV, cerebrovascular and renal diseases, which leads to a reduction in CV morbidity and mortality [2]. Therefore, the target goals for BP treatment in HTN have gradually lowered in recent decades. HTN was recently defined as systolic BP over 140 mmHg and diastolic BP over 90 mmHg, and patients with BP above these values should be treated [3]. To reach these treatment goals, a combination of different antihypertensive medications has proven to be most beneficial for patients, although ~60% are not adequately treated [4]. Moreover, there is a growing patient population not responding adequately to current antihypertensive therapy [5]. Therapy-resistant HTN (rHTN), as defined by the American Heart Association, includes patients who show an increased BP above the goal after having been treated with 3 antihypertensive agents of different classes or patients requiring 4 antihypertensive agents [6]. ESH/ESC guidelines define resistance to treatment if a therapy includes appropriate lifestyle measures, two different classes of antihypertensive drugs and a diuretic, all at adequate doses. If this regimen fails to lower systolic BP (SBP) and diastolic BP (DBP) to <140 mmHg and < 90 mmHg, respectively, a patient is classified as having rHTN [7]. The overall prevalence in hypertensive patients is estimated to range between 8% and 12%, potentially totaling 6–12 million people in the US alone [8, 9]. Several studies have shown that rHTN is a continuously growing problem. Egan and colleagues examined 13,375 hypertensive patients (NHANES; National Health and Nutrition Examination Survey) and found an increase in rHTN from 16% (1998–2004) to 28% (2005–2008) [8]. rHTN can be real, spurious or only apparent. The latter is often related to poor adherence to therapy, which is a common phenomenon in hypertension [10]. However, despite compliance-related rHTN patients, ‘real’ rHTN is present in half of rHTN patients [11]. Current pharmacologic treatment options for rHTN in addition to standard antihypertensive therapy include alpha blockers [12], direct renin inhibitors [13, 14], central sympatholytics, direct vasodilators [15] and mineralocorticoid receptor antagonists [16]. Spironolactone, which has been intensively studied in rHTN, is currently the only drug that has consistently shown benefits when added to standard of care (SoC) hypertensives in rHTN patients [17, 18]. However, as shown by epidemiologic data, these therapeutics do not or do not fully meet the unmet medical needs of the patient population affected by rHTN. In addition, surgical invasive treatments, such as renal denervation, have been studied clinically, with limited success [19]. The limitations of current treatment approaches still provide ample opportunities for novel therapies with improved properties addressing the well-recognized gaps.

**Fig. 1:**
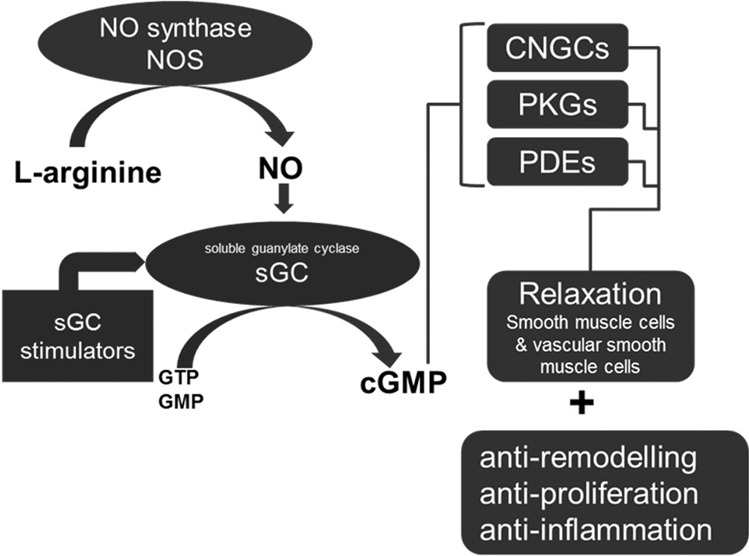
Mode of action of sGC stimulators (SGC, soluble guanylate cyclase; NO nitric oxide, cGMP cyclic guanylyl monophosphate, CNGC cyclic nucleotide–gated ion channel, GTP guanosine-triphosphate, GMP guanosine monophosphate, PKG protein kinase G, PDE phosphodiesterase)

The nitric oxide (NO)-soluble guanylyl cyclase (sGC)-cGMP signal transduction pathway is one of the most important regulators of vascular tone. As illustrated in Fig. 1, NO binds to the sGC enzyme, thereby stimulating cGMP production to cause the relaxation of vascular smooth muscle cell tone. Exploiting this mode of action, NO donors have been used since the 19th century and are approved for the treatment of angina pectoris. However, NO donors have a limited therapeutic window, and their utility is further restricted by their pharmacokinetics and tachyphylaxis. More recently, a new class of drugs, so-called sGC stimulators, was introduced. These compounds stimulate sGC and act synergistically with endogenous NO [20]. Riociguat (BAY 63-2521) is a potent and selective sGC stimulator and is a first-in-class sGC stimulator approved for the treatment of pulmonary arterial hypertension (PAH) and chronic thromboembolic pulmonary hypertension (CTEPH) [21]. In addition, it was shown that sGC stimulators such as riociguat and BAY 41-2272 or BAY 41-8543 lead to a dose-dependent BP reduction [22]. In healthy volunteers, sGC stimulation resulted in decreased mean arterial BP [23]. More recently, mutations in components of the NO/sGC pathway, especially in the sGC gene (GUCY1A3), have been linked to an increased CV risk, including myocardial infarction, coronary artery disease and HTN [24]. These data imply a pivotal role of sGC signaling in the regulation of vascular tone and endothelial and platelet activation and render sGC stimulators optimal drug candidates to treat HTN.

In our study, we therefore aimed to establish an animal model that is characterized by HTN that does not adequately respond to current HTN therapy reflecting (at least in part) rHTN. In addition, we investigated whether the sGC stimulator BAY 41-2272 is able to lower BP in this rHTN model, either as standalone treatment or in addition to SoC antihypertensives.

## Methods

### Study protocol

**Fig. 2:**
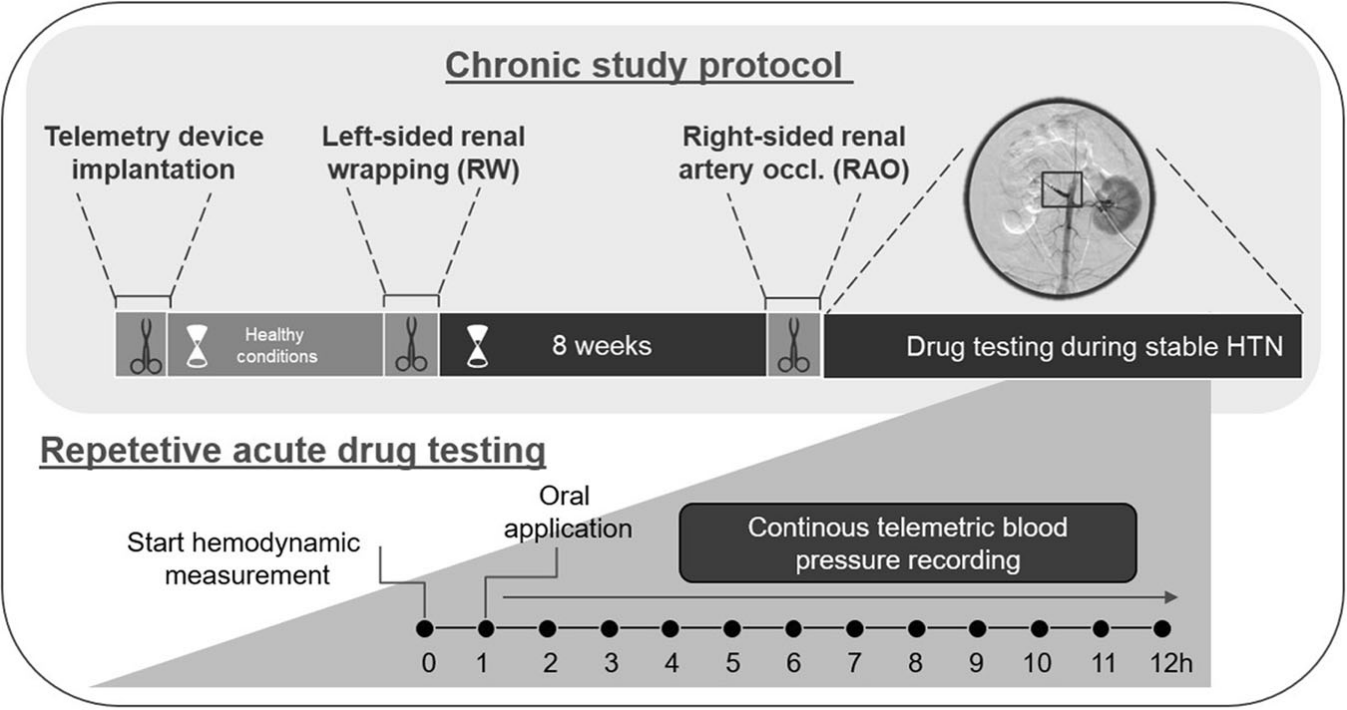
Study protocol

The overall study protocol is illustrated in Fig. 2. Six male beagle dogs (Marshall BioResources, USA) weighing between 10 and 15 kg were included in this study to evaluate the effect of BAY 41–2272. BAY 41‐2272 (3-(4-amino-5-cyclopropylpyrimidin-2-yl)−1-(2-fluorobenzyl)−1H-pyrazolo[3,4-b]pyridine) (Supplementary Fig. 1) is a typical member of the sGC stimulator compound family [25, 26]. BAY 41–2272 was given alone and in combination with the SoC. In the first surgery, animals were instrumented with telemetry sensors to evaluate hemodynamics in conscious dogs. In a second and third intervention, HTN was induced by left-sided renal wrapping (RW) followed by right-sided renal artery occlusion (RAO). After reaching a stable phase of HTN, repetitive acute drug testing was initiated to evaluate BP effects after once daily oral administration of BAY 41-2272, SoC antihypertensives or their combinations. Every single pharmacologic treatment was given on a single day, and the animals were monitored for the following 12 h. The overarching therapeutic goal was to decrease BP back to normal levels.

### Telemetry sensor implantation

For telemetry sensor implantation, animals were anesthetized with thiopental-sodium (Trapanal®, 0.25–0.5 mg/kg, Byk Gulden, Germany) and pancuronium bromide (0.20–0.25 mg/kg Pancuronium Inresa, Inresa Arzneimittel GmbH, Germany). After intubation, all dogs were mechanically ventilated with O_2_/N_2_O (1:3). Anesthesia was maintained with 1–2% isoflurane (Isoflurane Baxter, Baxter, Germany). For analgesia, fentanyl (10–40 µg/kg/h, Mallinckrodt Inc, USA) was infused via the right cephalic vein. After left site thoracotomy, a pressure sensor catheter (Model L21, Data Sciences International, USA) was implanted in the aortic vessel. For ECG measurement, the sensor contains 2 biopotential cables, which are positioned directly on the heart. After implantation, the skin and muscle sections were finally closed by Vicryl-suture (Ethicon, Johnson & Johnson, USA). All animals received enteral antibiotics (Clindamycin (Clerobe®), Zoetis/Germany; 150 mg/animal; p.o.) and Carprofen (Rimadyl®, p.o., 50 mg/animal, Zoetis, Germany) over a period of 10 days after sensor implantation. For additional analgesia, a Durogesic® patch (Fentanyl, 25 µg/h, Janssen-Cilag, Germany) was placed on the right thoracic side. Fourteen days postsurgery, the stitches were then removed.

### Renal wrapping

After wound healing, the left kidney was wrapped with silk in a second surgery. Therefore, the animals were anesthetized as described above. Under sterile conditions, the left abdominal cavity was opened. The left kidney was lifted and released from retroperitoneal tissue. Next, the kidney was wrapped with sterilized silk without applying excessive compression to the kidney. Subsequently, the kidney was dropped back into its bed. The peritoneum and all layers above, including the skin, were sutured using Vicryl-suture (Ethicon, Johnson & Johnson, USA). Medication after the surgical intervention was applied as follows: for antibiotic prophylaxis, enrofloxacin (Baytril® 5 mg/kg, Bayer Vital, Germany) was injected subcutaneously the first day and was administered orally (Baytril Flavor, 50 mg/10 kg, Bayer Vital, Germany) for the following 14 days. For analgesia, fentanyl patches (Durogesic®, 25 µg/h mg fentanyl, Janssen-Cilag, Germany) were applied for 3 postsurgical days. In addition, metamizole-sodium (50 mg/kg, Metamizol WDT, Wirtschaftsgenossenschaft deutscher Tierärzte eG, Germany), leveraging its antipyretic and analgesic effects, was administered i.m. on Day 1.

### Renal artery occlusion

Eight weeks post kidney wrapping, the contralateral (right) renal main artery was occluded by the insertion of a vascular plug (Amplatzer Vascular Plug II, St Jude Medical, USA). Anesthesia, intubation and mechanical respiration were performed as described above. The right common carotid artery was prepared for catheter intervention, and a vascular plug® was placed in the right renal artery to induce renal artery stenosis. To estimate the diameter of the renal artery and control for correct plug positioning, angiography (X-ray: Model Flexiview 8800, GE Healthcare; Contrast agent: Ultravist, Bayer, Germany) was used.

### Hemodynamic measurements

To evaluate the consequences of renal interventions on BP and heart rate, telemetric hemodynamic measurements (Data Sciences International, USA) were recorded once a week during the complete time course of the study. Measurements were conducted and averaged for 12 h (4 pm – 4 am the next day) under normal housing conditions. In addition to regular hemodynamic monitoring, telemetry recordings were performed to evaluate the hemodynamic response after oral administration of test compounds. Therefore, telemetric BP measurements were conducted, starting 1 h before drug administration and ending 11 h post administration. Data were processed and averaged over periods of 10 min.

### Biomarkers and clinical chemistry analysis

Plasma clinical chemistry and biomarkers of renal and heart function were measured. Blood samples were collected at healthy status 5 weeks after RW, 6 weeks after RAO, 39 weeks after RAO and 46 weeks after RAO in EDTA- and lithium-heparin tubes (S-Monovette®, lithium-heparin and EDTA, Sarstedt AG & Co, Germany). Clinical chemistry data (CK, creatinine kinase; Crea, creatinine; UA, uric acid; Urea; Prot, protein) were analyzed using a blood analyzer (Model ADVIA 2400, Siemens, Germany). Plasma renin activity (PRA) was analyzed using an RIA assay (DiaSorin, CA1533). Furthermore, plasma aldosterone levels were measured before renal interventions; 8 weeks post RW; and 4, 8, 12 16, 20, 24, and 28 weeks post RAO. Additional biomarkers were analyzed for the assessment of renal function (Cyst-C, cystatin C; NGAL neutrophil gelatinase-associated lipocalin), inflammation (MCP-1, monocyte chemoattractant protein-1) and cardiac remodeling (BNP, brain natriuretic peptide) using ELISAs (cystatin-C: canine cystatin-C, BioVendor, Cat# RD491009100R, Czech Republic; NGAL: Dog NGAL ELISA Kit, BioPorto, Cat# KIT 043, Denmark; MCP-1: canine CCL2/MCP-1, R&D Systems, Cat# CACP00, USA; BNP: RIA canine BNP assay, Phoenix Pharmaceuticals, USA).

### Echocardiography

To evaluate cardiac functionality and remodeling, repetitive transthoracic echocardiographic (Vivid I, GE Healthcare, USA) examinations were performed at healthy status, 8 weeks after RW, and 12 weeks and 26 weeks after RAO in anaesthetized animals. In addition to valvular function, ejection fraction (EF) and the left ventricular (LV) mass were determined. LV mass was analyzed using the American Society of Echocardiography formula [27]. All analyses were performed using EchoPAC™ software (GE Healthcare, USA).

### Medication, drug formulation and treatment regimen

Approximately 8 weeks after RAO, all animals achieved stable HTN, defined by a systolic value above 140 mmHg and a diastolic value above 80 mmHg. At this time point, the drug testing phase was initiated and took 50 weeks for all compounds and combinations.

At the beginning of the drug testing period and before every new drug or drug combination was tested, all six animals were given placebo-gelatinous capsules (only solvent) once to rule out a placebo effect. In general, each dose of all medications was given once on a single day, followed by a follow-up period of 11 h and a washout period of 7 days. All intended doses of one drug were tested in a dose-increasing regimen. Therefore, the characterization of an individual drug required 3–4 consecutive weeks, depending on the number of dose steps. The following drugs were included in the study: an ACE inhibitor (enalapril; 0.3 mg/kg, 1.0 mg/kg, 3.0 mg/kg, 10.0 mg/kg), an angiotensin II antagonist (valsartan; 3.0 mg/kg, 10.0 mg/kg, 30.0 mg/kg), a β-blocker (atenolol; 1.0 mg/kg, 3.0 mg/kg, 10.0 mg/kg), a calcium antagonist (amlodipine; 0.3 mg/kg, 1.0 mg/kg, 2.0 mg/kg), a diuretic (furosemide; 2.0 mg/kg, 4.0 mg/kg) and the sGC stimulator BAY 41-2272 (0.3 mg/kg, 1.0 mg/kg, 3.0 mg/kg). The respective doses were based on the recommendations of the American College of Veterinary Internal Medicine (ACVIM) [28]. According to this recommendation, dogs affected with HTN should benefit from the following drugs and doses: 0.5 mg/kg enalapril, 0.1–0.5 mg amlodipine, 0.25–1.0 mg/kg atenolol and 1.0–4.0 mg/kg furosemide. In our study, we titrated the respective doses when there was no or inadequate acute response on BP. It was further investigated whether combinations of drugs have substantial BP-lowering effects. Therefore, the following triple combinations were tested: enalapril (3.0 mg/kg) + furosemide (2.0 mg/kg) + amlodipine (1.0 mg/kg), valsartan (10.0 mg/kg) + furosemide (2.0 mg/kg) + amlodipine (1.0 mg/kg) and enalapril (3.0 mg/kg) + furosemide (2.0 mg/kg) + valsartan (10.0 mg/kg). To further evaluate whether the sGC stimulator BAY 41-2272 may have an additional effect in addition to a triple combination with established modes of action, BAY 41-2272 (1.0 mg/kg) was added to the following combination: furosemide (2.0 mg/kg) + valsartan (10.0 mg/kg) + enalapril (3.0 mg/kg). All drugs were formulated in 10% ethanol (ethanol 95% v/v, Fisher Scientific) and 90% polyethylene glycol 400 (CAS 202398–500 G, Sigma–Aldrich). The applied volume was 0.25 ml/kg. After formulation, the drug was transferred into gelatinous capsules for administration. In the case of drug combinations, the individual drug was dissolved and administered separately, which was accordingly considered in the corresponding placebo administration.

### Statistical analysis

The statistical analyses were performed using GraphPad Prism® software (Version 6.07, GraphPad Software Inc., USA). All data were expressed as the mean ± SEM or SD. For statistical analysis of drug-related BP effects, mean BP (MBP) values were averaged between 1 h and 12 h postadministration and compared to averaged BP values from corresponding baseline values (preadministration, 0–1 h). Statistical analysis of drug effects as well as consequences of renal interventions on BP, biomarkers and echocardiographic parameters were performed using a one-way ANOVA test corrected for multiple comparisons using Dunnett’s test. Statistical significance was defined as *p* ≤ 0.05.

## Results

The major aim of this study was to generate a model for the investigation of sGC stimulators for either standalone or combinational treatment approaches in rHTN. To this end, we employed a dog model based on severe renal impairment and concomitant sterile inflammation through RW combined with RAO. All 6 animals included in the study developed long-term stable hypertonus that was resistant to treatment with guideline antihypertensives, including beta-blockers, ACE inhibitors, diuretics and Ca antagonists.

### Characterization of the newly established dog model

#### Effect of RW and RAO on BP and cardiac hemodynamics

**Fig. 3:**
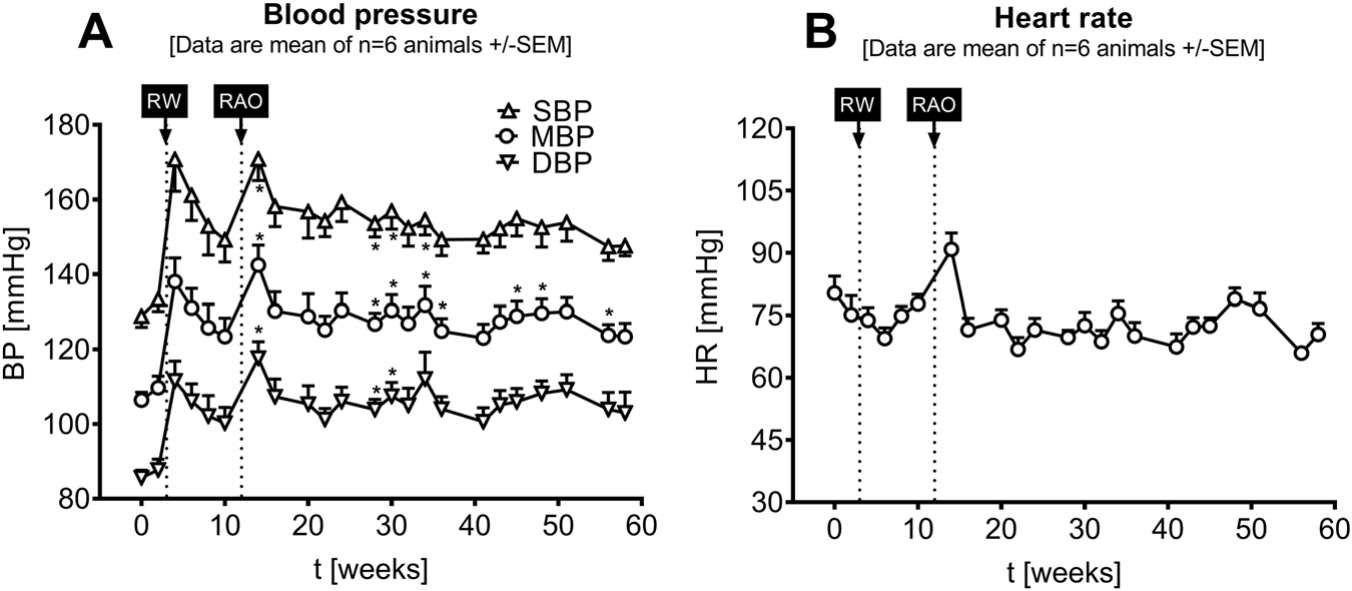
BP (Fig. 3A) and heart rate (HR) (Fig. 3B) development before and after renal interventions. Individual data points represent averaged data over 12 h between 4 pm and 4 am ± SEM. *p < 0.05, compared to healthy status

As shown in Fig. 3A, following device implantation prior to RW (‘healthy state’), all animals were normotensive with an MBP of 108.1 ± 3.9 mmHg. Three weeks after RW, animals developed a significant rise in MBP of +21.5 ± 8.3 mmHg on average (pre-RW versus weeks 4–10, *p* = 0.0255). Consecutive RAO at week 12 did not further increase BP; however, animals maintained stable and significant (*p* = 0.0070) HTN with an average (weeks 14–58) elevation in MBP of +20.4 ± 9.9 mmHg from baseline (pre-RW). Heart rate (Fig. 3B) was elevated shortly after RAO was performed from 77.7 ± 4.4 beats per minute (bpm, pre RW) to 90.8 ± 9.7 bpm (Week 14) but stayed constant over time at 72.7 bpm ± 5.4 bpm (weeks 14–58).

#### Biomarker and clinical chemistry assessment

As summarized in Table 1, additional biomarker analyses were performed to monitor the induced kidney disease and to diagnose inflammatory processes and possible end organ damage. BNP, a marker of cardiac remodeling, increased by +23.4 ± 15.3 pg/ml (6 wks post RAO, *p* = 0.0374) after kidney injury but returned to normal values at the end of the observation period. To evaluate the consequences of renal interventions on the renin-angiotensin-aldosterone system, PRA and aldosterone were measured. In comparison to healthy animals, PRA was elevated at a single timepoint 30 weeks post RAO (+0.33 ± 0.20 ng/ml/h, *p* = 0.0257) but was otherwise within the normal range. As shown in Supplement Fig. 2, plasma aldosterone levels increased from 57.3 ± 35.0 pmol/l to 117.8 ± 19.1 (8 wks post RW, *p* = 0.0966) after RW and further to 412.0 ± 316.4 pmol/l (*p* = 0.1978) 4 weeks after RW. However, there was only a single timepoint after 24 weeks post RAO at which aldosterone was significantly elevated (149.5 ± 47.0 pmol/l, *p* = 0.0363). MCP-1, a biomarker of the cellular inflammatory response, showed a trend toward higher levels after renal interventions, but this effect was significant (*p* = 0.0464) only 6 weeks post RAO. The same is true for cystatin C (CYSTC), a biomarker of glomerular filtration rate. In comparison to previous values in the healthy state, CYSTC was elevated by +764.0 ± 399.0 ng/ml 6 weeks post RAO (*p* = 0.0157). However, NGAL, also a predictor of acute kidney injury, was not significantly affected by RW or RAO but showed a trend toward higher levels after renal interventions.

**Table 1:** Biomarker and clinical chemistry data at several time points during the time course of the study

Plasmamarker	Healthy	Hypertension			
	pre RW	5 wks post RW	6 wks post RAO	30 wks post RAO	46 wks post RAO
No. of animals	6	6	6	6	6
BNP [pg/ml]	22.0 ± 5.9	35.2 ± 19.9	45.4 ± 10.9*	34.3 ± 9.9	25.6 ± 7.8
PRA [ng/ml/h]	0.33 ± 0.19	0.88 ± 0.93	0.36 ± 0.15	0.67 ± 0.21*	0.25 ± 0.10
MCP-1 [ng/ml]	129.50 ± 69.88	172.80 ± 58.77	213.20 ± 67.65*	149.00 ± 31.41	152.70 ± 34.48
NGAL [pg/ml]	40.98 ± 19.98	51.18 ± 30.21	47.48 ± 32.54	61.88 ± 51.28	73.33 ± 50.90
CYSTC [ng/ml]	1155.0 ± 368.5	1264.0 ± 193.7	1919.0 ± 384.4*	1677.0 ± 511.8	1715.0 ± 496.8
CK [U/l]	73.67 ± 22.82	105.80 ± 19.38	122.80 ± 45.39	110.30 ± 17.48*	107.30 ± 12.09*
Crea [µmol/l]	74.83 ± 5.78	86.00 ± 8.92	134.00 ± 33.21*	118.30 ± 29.00*	114.00 ± 25.65*
UA [µmol/l]	11.33 ± 1.51	15.17 ± 3.25*	14.33 ± 3.20	11.67 ± 1.97	14.17 ± 2.79*
UREA [mmol/l]	5.67 ± 0.84	7.26 ± 1.08	10.29 ± 3.68	8.44 ± 2.85	10.42 ± 2.93*
PROT [g/l]	59.53 ± 1.91	63.37 ± 3.67	62.62 ± 2.72	59.02 ± 2.31	58.53 ± 2.95

*BNP* brain natriuretic peptide, *PRA* plasma renin activity, *MCP-1* monocyte chemoattractant protein-1, *NGAL* neutrophil gelatinase-associated lipocalin, *CYSTC* cystatin C, *CK* creatinine kinase, *Crea* creatinine, *UA* uric acid, *Prot* protein

Data are expressed as mean of *n*  =  6  ±  SD. *p  <  0.05; ^†^*p*  <  0.01

To further characterize renal function, clinical chemistry data were obtained. CK, Crea, UA, UREA and protein (PROT) plasma levels were determined over the time course of the study. CK levels were elevated 30 weeks (+43.50 ± 27.65 U/l, *p* = 0.0150) and 46 weeks (+39.17 ± 23.67 U/l, *p* = 0.0182) post RAO and Crea plasma levels raised significantly from initial 74.83 ± 5.78 µmol/l to 134.00 ± 33.21 µmol/l after 6 weeks post RAO (*p* = 0.0179), 118.30 ± 29.00 µmol/l (0.0341) after 30 weeks post RAO and to 114.00 ± 25.65 µmol/l after 46 weeks post RAO. The measurement of UA showed a similar trend, whereas a significant increase in plasma levels was observed 5 weeks post RW (+3.83 ± 2.56 µmol/l, *p* = 0.0412) and 46 weeks (+2.83 ± 1.94 µmol/l, *p* = 0.0451) post RAO. Plasma UREA was elevated at a single time point 46 weeks after RAO ( +4.75 ± 3.29 mmol/l, *p* = 0.0470). However, plasma PROT levels were not significantly affected by renal interventions.

### Echocardiography

To evaluate potential changes in cardiac remodeling and hemodynamics before and after renal intervention, echocardiographic investigations were performed. As shown in Supplementary Fig. 3A, no differences in EF were observed in the time course of the study. However, LV mass (Supplementary Fig. 3B) significantly increased from 32.51 ± 1.25 g/m^2^ in the healthy group to 34.63 ± 1.11 g/m^2^ (*p* = 0.0106) 12 weeks after RW and further increased to 39.67 ± 3.56 g/m^2^ (*p* = 0.007) 26 weeks after RAO.

### Effects of drug monotherapy on BP in hypertensive dogs

**Fig. 4:**
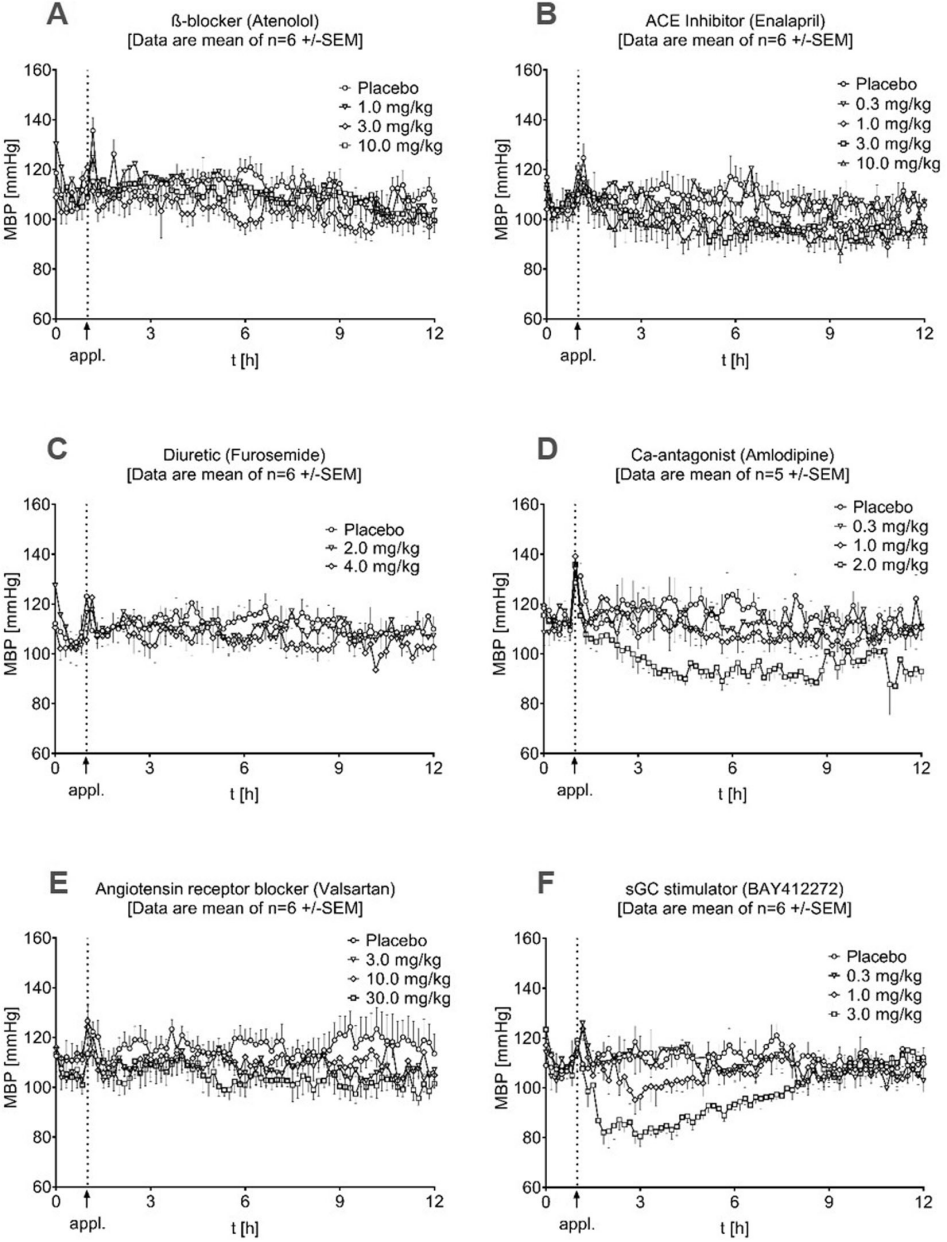
BP effects of drug monotherapies in hypertensive dogs. Individual data points are represented as the mean values of *n* = 6 ± SEM

Since the newly established dog model resulted in a stable HTN phenotype, we further characterized the animal model by single drug therapy using a broad range of clinically relevant antihypertensive treatments and the sGC stimulator BAY 41-2272. No difference in the baseline BP values (before administration) was observed in any of the dose-response curves (Fig. 4). With this, the same baseline conditions existed for all drugs and doses.

As already described, for drug characterization, we compared the averaged MBP values between 1 h and 11 h after the administration of individual drugs and respective doses to the individual averaged baseline values (0  – 1 h). For statistical analysis, we compared these data to the effects of the corresponding placebo group. We started using the beta-blocker atenolol (Fig. 4A), which led to a mean decrease in MBP of −6.57 ± 5.11 mmHg (*p* = 0.0545) at a dose of 1.0 mg/kg, a significant decrease of −7.22 ± 5.85 mmHg (*p* = 0.0394) at 3.0 mg/kg and a slight increase of 0.53 ± 7.50 mmHg (*p* = 0.7680) at 10.0 mg/kg.

Subsequently, medications addressing the renin-angiotensin system were tested. For enalapril (Fig. 4B), an ACE inhibitor, we observed a significant (*p* = 0.0125) effect of −7.88 ± 2.14 mmHg at the 1.0 mg/kg dose, −11.90 ± 4.53 mmHg (*p* = 0.0003) after 3.0 mg/kg and −8.75 ± 5.91 mmHg (*p* = 0.0057) after treatment with 10.0 mg/kg enalapril. The dose of 0.3 mg/kg (−0.06 ± 5.12 mmHg, *p* = 0.9994) had no significant effect compared to placebo (+0.74 ± 4.64 mmHg).

We further tested the loop diuretic furosemide (Fig. 4C) at doses of 2.0 and 4.0 mg/kg. None of the tested doses (2.0 mg/kg: −1.58 ± 6.23 mmHg, *p* = 0.4882; 4 mg/kg: +0.87 ± 7.62 mmHg, *p* = 0.8756) was able to adequately lower BP. In contrast, the calcium channel blocker amlodipine (Fig. 4D) led to a significant (*p* = 0.0021) decrease in MBP of −20.8 ± 14.18 mmHg at the highest dose of 2.0 mg/kg, whereas the 0.3 mg/kg (−4.75 ± 9.18 mmHg, *p* = 0.6056) and 1.0 mg/kg doses (−9.24 ± 6.32 mmHg, *p* = 0.1837) did not cause any significant effect. As shown in Fig. 4E, we further tested the angiotensin II receptor antagonist valsartan. Here, we observed a small decrease in MBP at 10.0 (−3.62 ± 5.84 mmHg, *p* = 0.0313) and 30.0 mg/kg (−4.06 ± 4.65 mmHg, *p* = 0.0222). However, the lowest dose of 3.0 mg/kg (+1.02 ± 3.39 mmHg, *p* = 0.5879) did not reach a significant difference compared to placebo.

The sGC stimulator BAY 41-2272 (Fig. 4F) had profound dose-dependent effects on MBP, with a decrease of −0.29 ± 5.40 mmHg (*p* = 0.7960) at the lowest dose of 0.3 mg/kg, −5.51 ± 11.45 mmHg (*p* = 0.1625) after 1.0 mg/kg and −14.05 ± 1.81 mmHg (*p* = 0.0042) after 3.0 mg/kg. It is important to note that some standard medications reported here were active only at doses significantly higher than reported therapeutically active doses.

### Effects of drug combinations on BP in hypertensive dogs

**Fig. 5:**
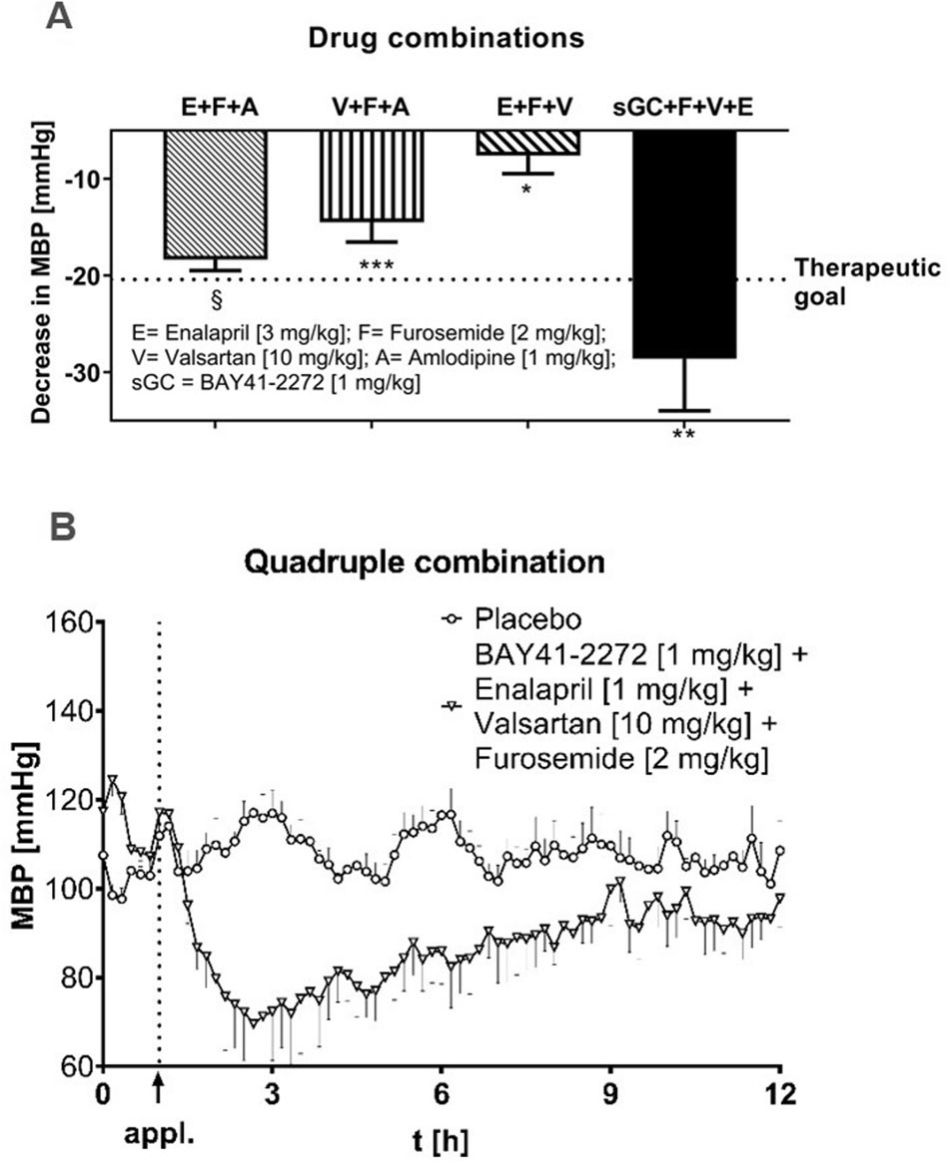
BP effects of drug combinations in hypertensive dogs. Data are represented as the mean values of *n* = 6 ± SEM. For statistical analysis, values were averaged between 1 h and 12 h after administration and compared to averaged values from corresponding baseline values (preadministration, 0–1 h); *p < 0.05, **p < 0.01, ***p < 0.001, § *p* < 0.0001

Following treatment guidelines for antihypertensive therapy, several triple combinations were subsequently investigated. As shown in Fig. 5A, the triple combination of enalapril (3.0 mg/kg), furosemide (2.0 mg/kg) and amlodipine (1.0 mg/kg) could decrease the MBP by −18.34 mmHg ± 2.75 mmHg, which was highly significant in comparison to placebo effects (*p* = 0.000073) but narrowly missed the therapeutic goal of −20.4 mmHg (averaged MBP increase induced by renal interventions, pre RW versus week 14–58). The second tested triple combination included valsartan (10.0 mg/kg), furosemide (2.0 mg/kg) and amlodipine (1.0 mg/kg). Here, we observed a moderate BP effect of −14.46 ± 5.05 mmHg (*p* = 0.00075).

A further triple combination tested in this model was the combination of enalapril (3.0 mg/kg), furosemide (2.0 mg/kg) and valsartan (10.0 mg/kg). The treatment of hypertensive animals with this combination resulted in an MBP decrease of −7.58 ± 4.58 mmHg (*p* = 0.01538), and in comparison to other combinations, it was classified as mildly effective.

The quadruple combination (Fig. 5A, B) of the sGC stimulator BAY 41-2272 at a dose of 1 mg/kg and the standard triple combination of enalapril (3.0 mg/kg), furosemide (2.0 mg/kg) and valsartan (10.0 mg/kg) showed a profound effect on MBP and exceeded the therapeutic goal. In comparison to baseline values (before administration), the combination of BAY 41-2272 (1.0 mg/kg), enalapril (3.0 mg/kg), furosemide (2.0 mg/kg) and valsartan (10.0 mg/kg) led to a significant (*p* = 0.00323) decrease in MBP of −28.59 ± 13.18 mmHg.

## Discussion

Most established canine models of surgically induced HTN are based on methods described by Goldblatt and Page [29, 30]. The model developed by Goldblatt is based on the constriction of renal arteries, whereas the Page model uses a cellophane- or silk-wrapped kidney. After an initial increase in BP, both models show a decline in BP over time and seem not to be suited for the investigation of chronic HTN on its own. Therefore, we aimed to develop a new model with stable but also therapy-resistant hypertension (rHTN). We combined both disease stimuli by consecutive interventions. Through the implantation of telemetry sensors, BP in these conscious dogs could be measured in a long-term follow-up with high temporal resolution. As shown in Fig. 3A, we demonstrated that the combination of RW and RAO led to a robust and long-term BP increase without any appreciable recovery over the entire observation period of 60 weeks.

One interesting feature of this dog CKD model is the low-renin phenotype. Neutral or decreased levels of renin have been as frequently observed across patients with rHTN [31, 32]. Nevertheless, from clinical studies, it is known that the two main mechanisms leading to HTN are sympathetic activation and aldosterone excess with concomitant fluid retention, which also provides the basis for MRI use in rHTN [33]. Therefore, it will be important in the future to study this preclinical phenotype in more detail and correlate findings with known features of human disease.

However, the major goal of our study was (a) to investigate whether SoC antihypertensives fail in this model, which could then be useful to study new drugs for rHTN treatment, and (b) to determine whether sGC stimulators could lower blood pressure in this rHTN as standalone therapy and in combination with SoC antihypertensives. Therefore, we started with an investigation of different antihypertensive treatments as standalone therapy. Only the calcium antagonist amlodipine showed an effect in the same range as the sGC stimulator (Fig. 4D). Thus, SoC hypertensives, even at higher doses, failed to reduce blood pressure to goal values. The reasons for these treatment failures might vary and could include the low-renin phenotype of the model (enalapril, valsartan) and the acute experimental study design. Plasma aldosterone measurement did not provide clear information; therefore, in future studies, a mineralocorticoid receptor blocker might be useful. However, in essence, these data are in line with clinical experience with standalone treatments that are not effective or are borderline effective. Thus, combinations were used, and within the tested triple combinations, the combination of enalapril, furosemide and amlodipine was most effective and only just missed the therapeutic goal. In comparison, the combination of valsartan, furosemide and amlodipine was less effective but had at least decreased MBP by almost 15 mmHg. However, we showed that in the single treatment group, the sGC stimulator BAY 41-2272 had a significant blood pressure-lowering effect in our disease model (Fig. 4F). The duration of the BP-lowering effect reflects the limited pharmacokinetic properties of this tool compound rather than being related to the efficacy of sGC stimulators in rHTN. With a view to the treatment of HTN alone, one may argue that amlodipine shows similar effects. However, this therapy also carries substantial side effects, such as peripheral edema, headache and facial flushing, among others, and in our study, amlodipine was primarily active in supra-efficacious doses.

In contrast, the combination of enalapril, furosemide and valsartan achieved similarly pronounced effects as enalapril alone and is therefore to be classified as less effective. Significantly more effective was BAY 41-2272 in addition to the triple combination of enalapril, valsartan and furosemide. In this quadruple combination, a BP-lowering effect of almost 30 mmHg was reached and thus even exceeded the previously defined therapeutic goal. In all investigations, no side effects were observed for any drug or combination, and a similar response to the tested drugs was observed in all animals included in our study.

In summary, we showed that dogs with unilateral renal wrapping combined with contralateral renal artery occlusion developed HTN that could be decreased only by common antihypertensives to a certain degree. In contrast to SoC antihypertensives, BAY 41-2272 caused a dose-dependent and stable blood pressure reduction in this model when given alone but also when dosed in addition to antihypertensive therapy. These data demonstrate that sGC stimulators might overcome the limitations of currently used antihypertensives. Thus, sGC stimulators might offer a treatment alternative for rHTN patients.

## Supplementary Infromation

Supplementary Figure 1
Supplementary Figure 2
Supplementary Figure 3
