# Supplementary figures and images for: sGC stimulation lowers elevated blood pressure in a new canine model of resistant hypertension
Source: Hypertens Res. 2021 Sep 22;44(12):1568–77. doi: 10.1038/s41440-021-00748-5 (PMC8645476; doi:10.1038/s41440-021-00748-5)

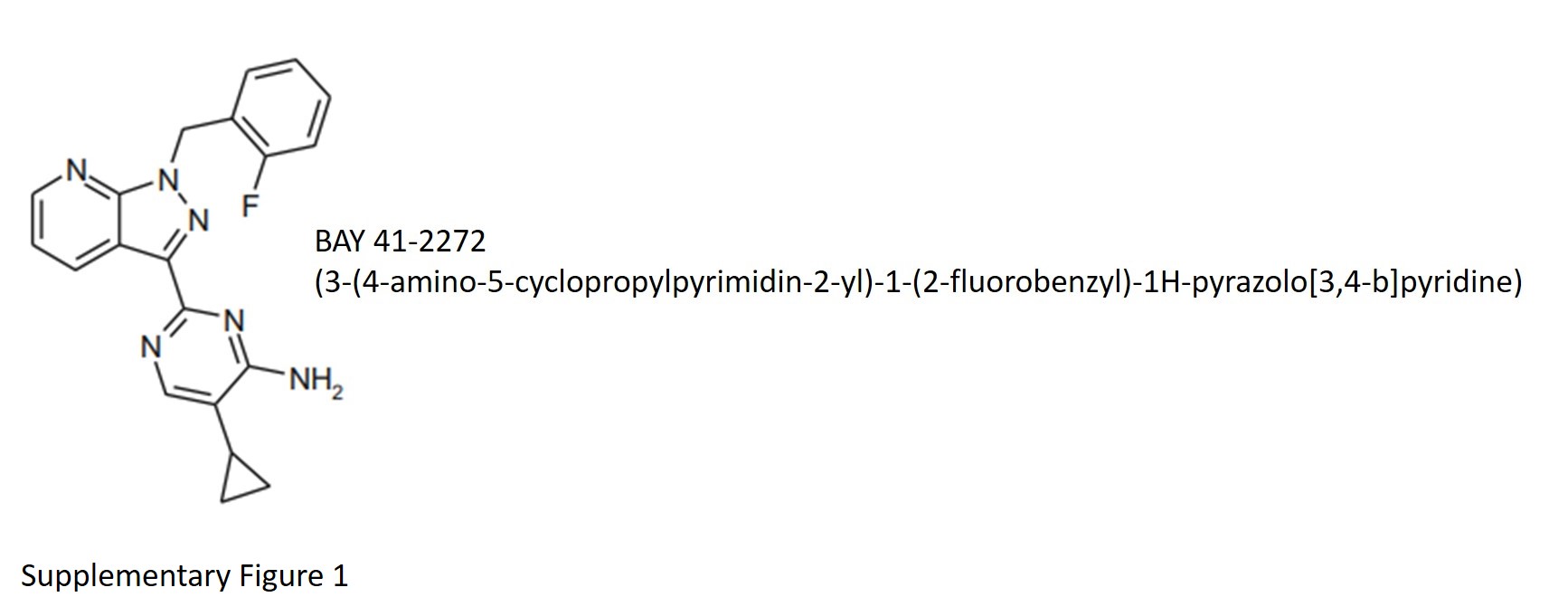

Supplement: Supplementary file 1 — Supplementary Figure 1 [file 41440_2021_748_MOESM1_ESM.jpg]

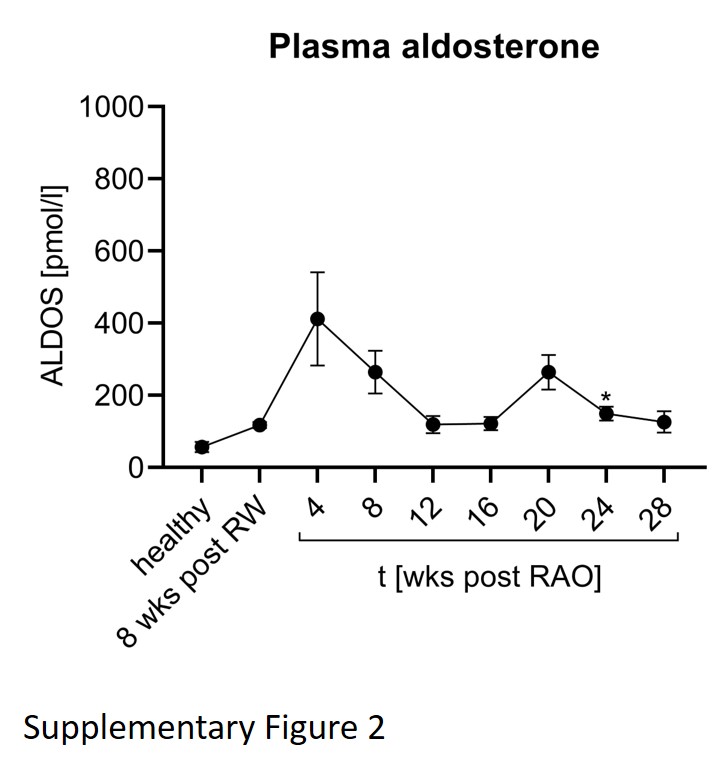

Supplement: Supplementary file 2 — Supplementary Figure 2 [file 41440_2021_748_MOESM2_ESM.jpg]

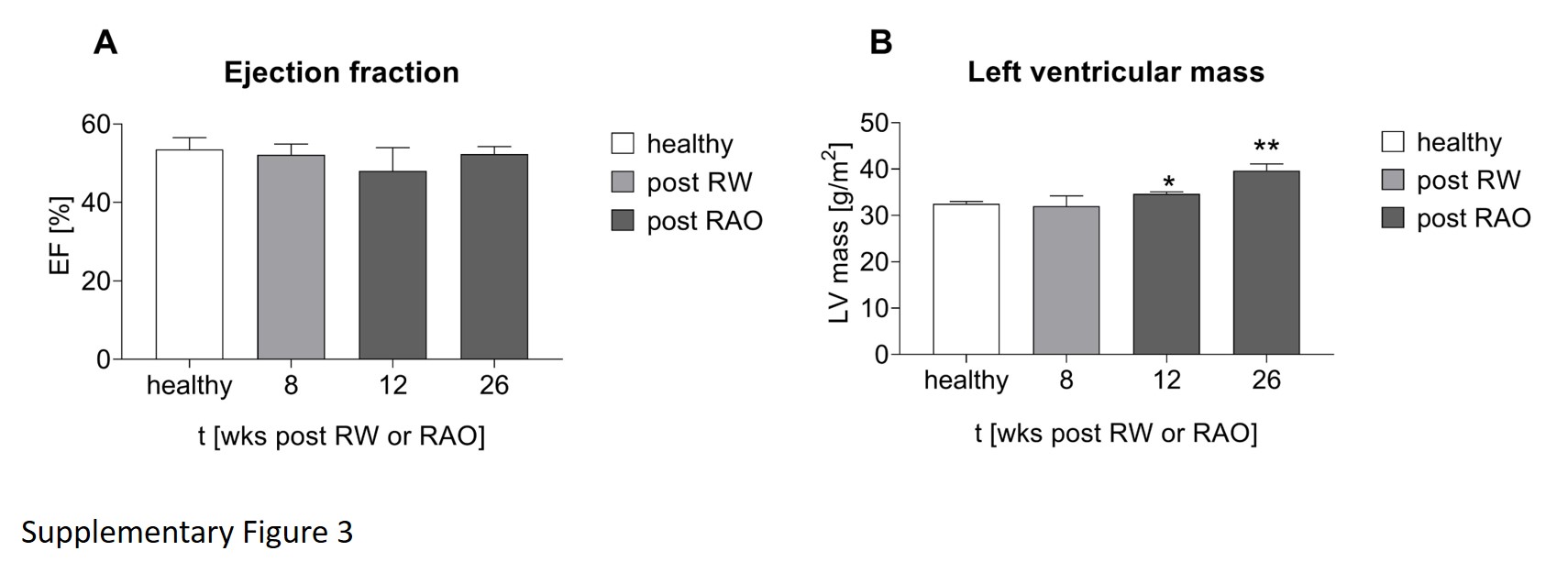

Supplement: Supplementary file 3 — Supplementary Figure 3 [file 41440_2021_748_MOESM3_ESM.jpg]
